# Conceptual control across modalities: graded specialisation for pictures and words in inferior frontal and posterior temporal cortex

**DOI:** 10.1016/j.neuropsychologia.2015.02.030

**Published:** 2015-09

**Authors:** Katya Krieger-Redwood, Catarina Teige, James Davey, Mark Hymers, Elizabeth Jefferies

**Affiliations:** Department of Psychology and York Neuroimaging Centre, University of York, UK

**Keywords:** Cognitive control, Semantic, Executive, Conceptual, Modality, Verbal, Picture, fMRI

## Abstract

Controlled semantic retrieval to words elicits co-activation of inferior frontal (IFG) and left posterior temporal cortex (pMTG), but research has not yet established (i) the distinct contributions of these regions or (ii) whether the same processes are recruited for non-verbal stimuli. Words have relatively flexible meanings – as a consequence, identifying the context that links two specific words is relatively demanding. In contrast, pictures are richer stimuli and their precise meaning is better specified by their visible features – however, not all of these features will be relevant to uncovering a given association, tapping selection/inhibition processes. To explore potential differences across modalities, we took a commonly-used manipulation of controlled retrieval demands, namely the identification of weak vs. strong associations, and compared word and picture versions. There were 4 key findings: (1) Regions of interest (ROIs) in posterior IFG (BA44) showed graded effects of modality (e.g., words>pictures in left BA44; pictures>words in right BA44). (2) An equivalent response was observed in left mid-IFG (BA45) *across* modalities, consistent with the multimodal semantic control deficits that typically follow LIFG lesions. (3) The anterior IFG (BA47) ROI showed a stronger response to verbal than pictorial associations, potentially reflecting a role for this region in establishing a meaningful context that can be used to direct semantic retrieval. (4) The left pMTG ROI also responded to difficulty across modalities yet showed a stronger response overall to verbal stimuli, helping to reconcile two distinct literatures that have implicated this site in semantic control and lexical-semantic access respectively. We propose that left anterior IFG and pMTG work together to maintain a meaningful context that shapes ongoing semantic processing, and that this process is more strongly taxed by word than picture associations.

## Highlights

• One of the first studies of semantic control across verbal and non-verbal modalities.
• Left mid-IFG supports controlled semantic retrieval for *both* words and pictures.
• Left and right posterior IFG show graded modality effects.
• Left anterior IFG and pMTG respond more to verbal than pictorial associations.
• Co-activation of these sites may maintain a context to shape semantic processing.

## 1 Introduction

Semantic cognition is inherently multimodal, allowing us to assign meaning to things we encounter in different modalities (words, pictures, actions, smells, etc.) and to map between these modalities (Jefferies, 2013; Lambon Ralph et al., 2008; Patterson et al., 2007). For example, on encountering the word “piano”, we can retrieve the full range of multimodal features for this concept, including visual properties (large size, black and white keys), actions (finger presses) and sounds (musical notes). Since only a subset of our semantic information is likely to be relevant for any given context, uncontrolled spreading activation within the conceptual store is insufficient for successful semantic cognition: we also need mechanisms that can focus processing on currently-relevant features or associations (Badre et al., 2005; Noonan et al., 2013b; Thompson-Schill et al., 1997). For instance, if you hear the question “how do you move a piano?” in the context of *moving house*, the dominant movements for the object (i.e., key presses) are irrelevant or even unhelpful (Saffran, 2000). Therefore, semantic cognition involves the interaction of (i) a store of multimodal semantic information, accessed by inputs in different modalities and (ii) control processes that shape semantic retrieval according to high-level goals established by the context or task (Jefferies and Lambon Ralph, 2006). However, the details of this interaction are still poorly understood. For example, there is next-to-no information in the literature about whether control processes vary across verbal and non-verbal tasks, because the vast majority of previous neuroimaging studies have presented written or spoken words (see Noonan et al., 2013b). Since concepts are multimodal, we might anticipate *identical* control processes for words and pictures; however, there are also differences in the information provided by these two types of inputs which might give rise to different executive demands, even when the task instructions are unchanged. Words have more flexible meanings and their interpretation is highly dependent on the context in which they appear. In contrast, pictures are more constrained by their visual features; however, not all of these features will be relevant in any given task, potentially increasing inhibitory requirements. In this study, we explored similarities and differences in the neural basis of controlled semantic retrieval from words and pictures.

Verbal and non-verbal semantic tasks elicit common activity in a large-scale cortical network that includes bilateral anterior lobes (ATL), left angular gyrus, bilateral inferior frontal gyrus (IFG), and left posterior temporal cortex with a peak for multimodal processing in posterior middle temporal gyrus (pMTG) (Adams and Janata, 2002; Binder et al., 2009; Bright et al., 2004; Chee et al., 2000; Vandenberghe et al., 1996; Visser et al., 2012; Visser and Lambon Ralph, 2011). However, neuropsychological, TMS and neuroimaging studies have revealed dissociations between brain regions implicated in (1) the representation of multimodal concepts (in ATL) and (2) the controlled retrieval of semantic information (in IFG and pMTG) (Jefferies and Lambon Ralph, 2006; Noonan et al., 2013b; Whitney et al., 2011a). Patients with semantic dementia following ATL atrophy show progressive *degradation* of conceptual knowledge: their deficits are highly consistent across different modalities (for example, across word, picture, sound and smell inputs) and performance is poorer for specific concepts and unique features (e.g., the camel’s hump is forgotten before the camel’s tail) (Bozeat et al., 2000; Lambon Ralph et al., 2010; Patterson et al., 2007; Rogers et al., 2004). In contrast, patients with semantic aphasia following left inferior frontal or temporoparietal stroke show *deregulated* semantic retrieval which is dominated by strong associations even when these are irrelevant to the task being performed. Their comprehension is strongly influenced by the degree of competition between concepts and the extent to which the task constrains semantic processing, reducing the need for internally-generated control (Corbett et al., 2009a; Gardner et al., 2012; Jefferies and Lambon Ralph, 2006; Jefferies et al., 2008; Noonan et al., 2010). While semantic aphasia patients have large lesions, neuroimaging and TMS studies of healthy participants also show effects of executive-semantic demands in LIFG and pMTG (Badre et al., 2005; Noonan et al., 2013b; Whitney et al., 2011a, 2011b). The literature therefore suggests that LIFG and pMTG work together to underpin controlled semantic retrieval, while ATL is involved in semantic representation (for review see Jefferies, 2013). This distinction is also broadly consistent with the findings of fMRI studies that have used multi-voxel pattern analysis to investigate brain areas representing specific semantic features and concepts: several studies using simple tasks without marked control demands have found that features and concepts can be classified by responses in ATL but not LIFG (Correia et al., 2014; Coutanche and Thompson-Schill, 2014; Peelen and Caramazza, 2012; but see Simanova et al., 2014).

A crucial question for this investigation concerns how this distributed functional system appropriately shapes semantic cognition for word and picture inputs. A recent activation likelihood estimation (ALE) meta-analysis examining tasks tapping semantic control in different ways (for example, understanding ambiguous words, retrieving distant semantic relationships or dealing with strong distractors) revealed a common response within bilateral IFG (extending to inferior frontal sulcus (IFS) and premotor cortex), left pMTG, dorsomedial prefrontal cortex (pre-SMA), and left dorsal angular gyrus (dAG) bordering on intraparietal sulcus (IPS) (Noonan et al., 2013b). These regions were commonly activated when semantic processing was difficult; however, the vast majority of these studies employed words (85% used verbal stimuli; 13% used a combination of words and pictures; with next-to-no studies examining picture-only tasks). The meta-analysis also explored which of these regions additionally showed activation for control-demanding phonological tasks: substantial overlap was seen in posterior parts of LIFG/premotor cortex, consistent with the view that these sites contribute to controlled aspects of linguistic processing more broadly, while anterior LIFG and pMTG showed a specifically semantic response (see also Gough et al., 2005; Wagner et al., 2001). Several of the sites identified by this meta-analysis – namely inferior frontal sulcus, IPS and pre-SMA – form a distributed frontoparietal control network supporting executive control across multiple domains and tasks (e.g., the multi-demand network described by Duncan, 2010; Duncan and Owen, 2000); it is therefore unsurprising that these regions are recruited during challenging conceptual tasks, and we would expect these regions to be recruited for more difficult judgements to both words and pictures. However, left anterior IFG and pMTG fall outside this multi-demand system (Blank et al., 2014; Fedorenko et al., 2012; Noonan et al., 2013b) and could conceivably show a differing response across input modalities or different types of tasks.

To develop hypotheses about the way in which the recruitment of semantic control regions might vary with modality, we first discuss the contribution that IFG and pMTG make to the executive control of semantic cognition in verbal tasks. The control of semantic cognition is multi-faceted: for example, in the case of goal-driven semantic control, attention can be focussed on specific aspects of knowledge according to the task instructions, while irrelevant features are suppressed (e.g., to answer the question “do pianos and penguins have the same colour?”, retrieval must be focussed on colour knowledge). Additionally, a representation of the current context (e.g., “we are moving house”) can be used to shape the conceptual response to inputs (e.g., on hearing the word “piano”, attention can be focussed on size and weight features, as opposed to its function as a musical instrument). Nevertheless, the meta-analysis of Noonan et al. (2013b) found that broadly the same network was recruited across tasks – and perhaps this is unsurprising since, in all cases, there is a need to use a goal or context to shape competitive processes such that relevant aspects of knowledge receive greater activation, and irrelevant information is supressed. Noonan et al. (2013b) suggested that co-activation of IFG and pMTG is crucial for the establishment, maintenance and efficient application of task, goal and context representations to ongoing conceptual processing, such that semantic cognition is biased towards aspects of meaning which are currently relevant. Nevertheless, we might still see graded specialisation of function within and between these regions (Badre et al., 2005; Noonan et al., 2013b). Below, we develop predictions about potential differences between words and pictures concerning (i) anterior and posterior IFG; (ii) left and right IFG and (iii) left IFG and pMTG.

*Anterior vs. posterior IFG*: Following the early debate about whether the contribution of LIFG to semantic cognition is best characterised as “selection” or “controlled retrieval” (Thompson-Schill et al., 1997; Wagner et al., 2001), Badre et al. (2005) suggested that *posterior* parts of LIFG are crucial for selection, while anterior parts of LIFG are more crucial for controlled retrieval. By this view, posterior IFG might bias competitive processes within the semantic system in favour of task- or context-relevant aspects of knowledge and away from strong distracters, while anterior IFG might be more important for establishing which aspects of conceptual knowledge should be the focus of ongoing processing (see also Bookheimer, 2002). This distinction is broadly consistent with proposals about the general organisation of cognitive control, which suggest that anterior parts of PFC maintain high-level goals which determine which features of representations are currently relevant for action or cognition, while posterior parts of PFC instantiate mechanisms that resolve competition between competing alternative responses (Badre and D’Esposito, 2009; Demb et al., 1995; Gabrieli et al., 1998). Since words occur in different contexts and have flexible meanings, we might hypothesise that generating a context that can provide a semantic link between two specific words is relatively demanding of anterior LIFG; in contrast, pictures are richer stimuli and their precise meaning is better specified by their visible features, which may reduce the response in anterior LIFG.

Despite these possible differences, there is a similar need to select targets and inhibit task-irrelevant relationships in both modalities, and this might drive a common response to words and pictures in more posterior parts of LIFG. Similarly, a common response might be seen for verbal semantic and phonological tasks, since both involve the selection of representations and responses. In line with this proposal, Snyder et al. (2007) suggested that phonological judgements to words generate more response conflict than those employing non-words (since participants must inhibit lexical-semantic retrieval): they manipulated selection demands for phonological judgements (via lexicality) and semantic decisions (based on global vs. feature-based similarity) and observed a common response in posterior LIFG to both manipulations. However, there are also potential differences in the control demands of semantic and phonological judgements, since semantic tasks often require participants to establish which aspects of knowledge are relevant for the current decision. This need to extract and maintain a meaningful context that can flexibly shape ongoing processing provides a potential explanation for the well-established finding of greater recruitment for semantic than phonological tasks in anterior LIFG (contrasting with posterior LIFG) (e.g., Badre et al., 2005; Devlin et al., 2003; Gough et al., 2005; Wagner et al., 2001; Whitney et al., 2011b).

*Left vs. right IFG*: While the meta-analysis of Noonan et al. (2013b) confirms that there is bilateral recruitment of IFG in response to executive-semantic demands, the response in this region is predictably stronger in the left hemisphere. This bias could reflect a special role for LIFG in conceptual processing across modalities, or alternatively might reflect the fact that the vast majority of the studies in this meta-analysis employed words (see above). Since functional connectivity between IFG and other brain regions involved in semantic processing is stronger within than across hemispheres, graded differences in the recruitment of left and right IFG for words and pictures could be driven by hemispheric differences in conceptual representations or the inputs to this system. In addition, there could be a division of labour between LIFG and RIFG in the contribution of these regions to specific aspects of cognitive control.

Semantic representations in the ATL are thought to be multimodal and differences between words and pictures are not strongly evident (Bozeat et al., 2000; but see Mion et al., 2010; Pobric, Jefferies and Lambon Ralph, 2010; Visser et al., 2012; Visser and Lambon Ralph, 2011); nevertheless, the specialised contribution of the left hemisphere to language processing is well-established, and there is a division of labour in occipital–temporal areas – with left posterior fusiform cortex showing a greater response to written words, and the right-hemisphere homologue showing a stronger response to faces (Cohen et al., 2002; Kanwisher et al., 1997). While these differences could produce some specialisation for word and picture tasks within left and right IFG, the neuropsychological evidence is mixed: there are reports of left hemisphere cases with semantic access deficits that are selective for verbal or auditory stimuli (Crutch and Warrington, 2008; Warrington and Crutch, 2004), yet patients with semantic aphasia following left IFG or pMTG lesions have *multimodal* semantic control deficits (Gardner et al., 2012; Jefferies and Lambon Ralph, 2006; Noonan et al., 2013a). Interestingly, patients with similar right hemisphere lesions tend not to show pronounced linguistic or semantic deficits, but they often have problems with high-level semantic tasks – such as drawing inferences and understanding metaphors presented as words and pictures (Brownell et al., 1986, 1990). Thus, the two hemispheres may make partially distinct contributions to semantic control: the left hemisphere may be more important for multimodal semantic cognition overall, while the right hemisphere establishes broad semantic relationships and supresses overly narrow ‘literal’ interpretations of metaphors (Jung-Beeman, 2005).

The proposal that left and right IFG make distinct contributions to semantic cognition remains highly controversial (Lee and Dapretto, 2006); however, the proposals above are compatible with a broader literature on executive control (Aron et al., 2004; Garavan et al., 1999). By this view, left PFC is specialised for the *selection* of specific representations or responses, while right PFC is driven by broader patterns of spreading activation, and plays a crucial role in inhibition (Aron et al., 2004; Chikazoe et al., 2007; Lenartowicz et al., 2011). This might drive differential recruitment of left and right IFG in semantic association tasks employing words and pictures. Words can be used in different ways, and their interpretation depends on the context: therefore, association-matching tasks employing these stimuli might require participants to *select* an appropriate interpretation or set of features that can link two words together. In contrast, pictures provide much richer information, including all of the concrete features of an object – however, since only a subset of these features are relevant in any given task, picture tasks may involve more inhibition.

*Left IFG vs. pMTG*: As noted above, there is increasingly strong evidence, across several methods (neuropsychology, TMS, fMRI) that LIFG and pMTG co-activate when executive-semantic demands are high. Lesions centred on either LIFG or pMTG in patients with SA give rise to similar deficits – namely, difficulty retrieving appropriate conceptual information in semantic tasks with high control demands (Jefferies and Lambon Ralph, 2006). TMS to either of these locations disrupts controlled semantic retrieval (i.e., judgements based on the selection of specific semantic features or the retrieval of weak associations) but not more automatic spreading activation between strongly-linked concepts (Whitney et al., 2011b). Moreover, the neuroimaging meta-analysis of Noonan et al. (2013b) shows reliable co-activation of these sites in contrasts targeting the executive demands or difficulty of semantic processing, even though this literature has largely only considered the role of LIFG.

pMTG is also unique within posterior temporal cortex in that it shows a multimodal response to semantic tasks employing either pictures or words (Visser et al., 2012); semantic decisions in both modalities are disrupted by TMS to this region (Hoffman et al., 2012). These findings fit well with the multimodal semantic control deficits seen in patients with SA following posterior temporoparietal lesions (Jefferies and Lambon Ralph, 2006; Noonan et al., 2010). On the basis of this evidence, we have proposed that the co-activation of LIFG and pMTG underpins the flexible and controlled retrieval of semantic information according to current goals or context, irrespective of whether concepts are accessed from pictures or words (Jefferies, 2013; Jefferies and Lambon Ralph, 2006). However, this hypothesis could not be tested within Noonan et al.'s (2013b) meta-analysis since very few neuroimaging investigations had manipulated semantic control demands for non-verbal stimuli.

Other researchers have variably suggested (i) that posterior temporal lobe regions are a key site for our repository of multimodal semantic representations (Kable et al., 2005; Kellenbach et al., 2003; Martin, 2007); (ii) that pMTG underpins (multimodal) event representations (Chao and Martin, 1999; Gennari et al., 2007; Humphreys et al., 2013; Kable et al., 2005) and (iii) that pMTG plays a crucial role in mapping from linguistic representations to multimodal concepts (Hickok and Poeppel, 2004, 2007). The proposal that pMTG forms a lexical interface underpinning conceptual retrieval from words has difficulty accounting for the multimodal response in this region. Moreover, these perspectives would apparently not predict the similar response seen in LIFG and pMTG to executive-semantic demands (Badre et al., 2005; Gold et al., 2006; Noppeney et al., 2004; Rodd et al., 2005; Whitney et al., 2011a; Zempleni et al., 2007). Badre et al. (2005) and Gold et al. (2006) both reported that pMTG showed a similar pattern of response to anterior LIFG – namely increased activation in conditions requiring more “controlled retrieval” – distinct from a “selection” response in posterior LIFG. In addition, Noonan et al. (2013b) found a bilateral response to executive-semantic demands in posterior IFG, while the response in anterior IFG and pMTG was left-lateralised. These findings taken together raise the interesting possibility that there may be two distinct yet strongly interacting systems contributing to semantic control in the brain: one system is bilateral and might overlap with domain-general executive mechanisms in posterior IFG/IFS; the other is predominately left-sided, less clearly overlapping with the fronto-parietal control system, and draws on the co-activation of anterior LIFG and pMTG.

In the present study, we contrasted easy and difficult semantic decisions to words and pictures, in order to determine whether different input modalities produce differential recruitment across brain regions that support executive semantic processing. Specifically, we investigated whether there are (i) functional specialisations within LIFG, (ii) hemispheric differences between left and right IFG and (iii) dissociations within temporoparietal regions (pMTG, dAG, vAG). We also examined the brain's response to executively-demanding linguistic but *non-semantic* decisions to establish which regions responding to semantic control also show a strong response to language control more generally. We anticipated a response to difficulty in posterior IFG across modalities and tasks, since the more difficult versions of all of the tasks required selection/inhibition. In addition, if there is a greater need to identify a linking context for verbal associations, this might be accompanied by stronger activation within a distinct anterior LIFG-pMTG network associated with controlled *semantic* retrieval specifically.

## 2 Method

### 2.1 Design

A within subjects 2×2 factorial design was used for the semantic tasks, with modality (verbal semantic, picture semantic) and difficulty (strong/easy vs. weak/harder associations) as factors. These tasks were compared with two other non-semantic yet linguistic decisions – a relatively easy rhyme judgement task, and a challenging phoneme segmentation task that involved problem-solving and working memory. This hard non-semantic task allowed us to establish which brain regions responding to semantic control contrasts were also activated by executive-demanding non-semantic judgements.

### 2.2 Participants

Data were acquired from twenty-three right-handed, native English speakers (16 males; mean age=23.2, SD=2.9). One participant with low accuracy (53%) was removed from the analysis. Participants were compensated £10 for their time.

### 2.3 Tasks

A three alternative forced choice (3AFC) format was used for all three tasks (see Table 1 for example stimuli). The non-semantic and verbal semantic tasks involved auditory presentation of a probe word, and response options presented as written words. The picture semantic task used photographs of the probes, targets and distracters.

The semantic tasks involved easy and hard associative judgements: participants were presented with a spoken word or picture probe, together with three word/picture response options on the screen. They were instructed to select the item most strongly related to the probe. The probes and targets either shared a strong association (for easy trials), or a weak association (for more difficult trials). For example, an easy association might involve the probe “duck”, and three answer choices such as lake–cigar–door. A harder trial would require participants to link “duck” with gun – an association that is less frequently encountered. Strong associations are thought to be retrieved relatively automatically, given that undirected spreading activation from the probe item is likely to rapidly activate the target. In contrast, for weak associations, the probe is likely to generate spreading activation in many irrelevant directions, and thus control processes are necessary to focus retrieval on non-dominant semantic features that are relevant to the linking context (e.g., a duck can be hunted; a gun is used for hunting). This manipulation of strength of association has been used previously by many neuroimaging studies comparing relatively automatic vs. more controlled forms of semantic retrieval (Badre et al., 2005; Wagner et al., 2001) and can be readily adapted for picture stimuli.

The easy non-semantic task required participants to make rhyme judgements: they heard an auditory probe such as “duck” and were required to choose the appropriate rhyming word from three on-screen choices (e.g., truck–cigar–game). Eye-rhymes were included to prevent participants from matching on the basis of orthography (e.g., “moon”–cigar–prune–game). The hard non-semantic task required participants to match a phoneme from the auditory probe word to the correct written target, which had the relevant phoneme missing. Participants performed this task in mini-blocks in which they were instructed as to which phoneme to pay attention to (i.e., “match first”, “match last”). The task consisted of an auditory probe and three answer choices (for example, a “match last” trial would consist of an auditory probe “duck” and three on-screen choices (e.g., tru_ – _ree – ga_), where matching “ck” to “tru” is the correct response (truck). Thus, the hard non-semantic task involved problem-solving and placed significant demands on working memory demands and meta-linguistic abilities. It was expected to elicit strong activation of the ‘multi-demand’ executive system, allowing us to establish whether brain regions responding to the contrast of hard>easy semantic judgements would also respond to *non-semantic* executive demands.

### 2.4 Stimuli

Stimuli were auditory probes in the non-semantic and verbal semantic conditions, and targets and distractors appeared on a black screen. Auditory probes were recorded by a male native English speaker, using Audacity (http://audacity.sourceforge.net/), in a sound-attenuated room. The stimuli were normalised for volume and power by digitally scaling them in Matlab (www.mathworks.co.uk/), producing a level of −25 dB FS. The MRI auditory stimulus system (MR Confon mkII+, www.mr-confon.de/en/products.html) was used to give a maximum presentation level of 80–90 dB SPL. The stimuli for the picture semantic task were coloured pictures sourced from the internet and fitted to a standard 255×149 pixel size using image manipulation software (GIMP: http://www.gimp.org/; Adobe Photoshop 7.0: www.adobe.com; ImageMagick 6.3.7.9: www.imagemagick.org/script/index.php).

Stimuli were all concrete nouns acquired from the MRC psycholinguistic database (concreteness and imageability>500; Coltheart, 1981; Wilson, 1988). Two websites (www.rhymezone.com and www.rhymer.com) were used to generate targets for the non-semantic easy task. The Edinburgh Association Thesaurus (http://www.eat.rl.ac.uk) was used to generate items for the semantic tasks but was not used for the final assignment of items into strong and weak association conditions, since verbal associative strength might not be applicable to picture stimuli: for example, the words ‘zebra’ and ‘crossing’ have a high verbal association as they are used together in the phrase ‘zebra crossing’, but photographs of these items would *not* be judged as being strongly linked. For this reason, we collected ratings of associative strength on a 5 point scale in a separate group of participants (*n*=48) not included in the fMRI study. These ratings were obtained separately for word and picture stimuli so that we could ensure our experimental manipulation of difficulty was equivalent across the two modalities (see Table 2).

There were 90 verbal semantic, 90 picture semantic and 90 phonological trials in total, and reaction time (RT) was recorded in the scanner for each trial. RT was explicitly modelled during fMRI analysis to account for effects of time on task (see below). There was a strong correlation between RT and rated associative strength for both picture (*r*=.89, *p*<.001) and word (*r*=.83, *p*<.001) semantic trials. Therefore, in order to maximise the difference between easy and hard semantic trials, the fastest and slowest one third (of the total accurate trials) were selected for analysis for each participant. There were no significant differences between the picture and word tasks overall in either RT (*t*(479)=−.532, *p*=.6) or rated associative strength (*t*(479)=.453, *p*=.65), treating items as cases. However, there were substantial differences in rated associative strength between the easy and difficult trials selected for analysis (see Table 2). We computed the average associative strength for the word and picture items within the ‘easy’ and ‘hard’ conditions for each participant and submitted these data to a within-subjects ANOVA, revealing a main effect of difficulty (*F*(1,21)=742.6, *p*<.001), no effect of modality (*F*(1,21)<1), and no interaction (*F*(1,21)=1.73, *p*=.2). To contrast these semantic decisions with easy and hard non-semantic decisions, trials from the rhyme and segmentation tasks were selected for each participant to match semantic RTs across easy and hard conditions. 24 trials for each condition were taken because this was the average number of trials used for each semantic condition for each participant.

The same probes were used across all tasks (non-semantic, verbal semantic, picture semantic) and for the easy and hard versions of each task; however, there were additional probes for the semantic tasks (given that there were two sets of semantic judgements). Unrelated distracters (two for each trial) were taken from other conditions within the experiment. The semantic associations were the same across verbal and picture semantic tasks, however, no trial was repeated across verbal and picture semantic conditions within a single subject.

Table 3 shows the Celex written frequency and rated imageability of the probe and target stimuli used in the fMRI analysis (Coltheart, 1981; Wilson, 1988). Log frequency did not differ significantly for the probes or targets used in easy and hard trials, across any of the judgement types (*F*(1,21)=1.93, *p*=.665), nor did it differ significantly across tasks (*F*(2, 42)=1.202, *p*=.311). Imageability was matched across semantic tasks (*F*(1, 21)=1.13, *p*=.3) but there was a small yet significant difference between the hard and easy semantic judgements selected for analysis (*F*(1, 21)=17.925, *p*<.001). Moreover, due to constraints on stimulus selection in the semantic and rhyme tasks, the items used in the phonological tasks were lower in imageability (*F*(1, 21)=137.282, *p*<.001) than those used in the semantic tasks. However, our objective was not to compare semantic and phonological processing directly (since this has been done previously); instead the comparison between the two phonological tasks allowed us to establish whether each brain region involved in the controlled retrieval of semantic information also responded to difficulty in a non-semantic linguistic task.

### 2.5 Procedure

A PC running Presentation 13.1 software (Neurobehavioural Systems, www.neurobs.com) was used to present the tasks and record accuracy and RT in the scanner. Responses were given with the left hand (such that motor responses would activate the right hemisphere), with three buttons corresponding to the positions of the three response options on the screen. The tasks started with a fixation screen for a jittered amount of time (500–2000 ms) followed by the trial (auditory probe and on-screen target and distracters). Participants were required to make a response, which triggered the next trial; if no response was given after 5 s the experiment moved onto the next trial.

The experiment began with a practice prior to entering the MRI scanner, to familiarise participants with the tasks (63 trials total). The experimental task consisted of 90 experimental trials per task type (phonological, verbal semantic, picture semantic), with participants performing a total of 270 trials. The tasks were presented in mini blocks of 15 trials per block, with a total of 18 blocks (6 blocks per condition). Each mini-block was followed by 7 s of rest, with a fixation cross on the screen. The order in which the trials occurred was pseudo-randomised and the order in which the tasks were presented was counterbalanced across participants. Each task block was preceded by a screen which informed participants of the new task type: the duration of this instruction screen was 1 s.

### 2.6 Image acquisition

Data were acquired with a GE 3 T HDX Excite MRI scanner at the York Neuroimaging Centre (YNiC), in a single scanning session. A Magnex, 8 channel, gradient insert head coil with a birdcage, radio frequency coil tuned to 127.4 MHz was used. A gradient-echo EPI sequence was used to collect data from 39 contiguous axial slices (TR 3 s, TE=25 ms, FOV 260 mm^2^, matrix size=128×128, slice thickness=3.5 mm). The functional data were co-registered onto structural T1-weighted images with a resolution of 1 mm×1 mm×1 mm (TR=8.03, TE=3.07 ms, FOV 290 mm×290 mm×176 mm, matrix size 256×256×176, slice thickness=1.13 mm×1.13 mm×1 mm). Functional data were additionally co-registered to T1 weighted FLAIR images (5.6 mm×5.6 mm×3.5 mm), taken in the same plane as the EPI slices with interleaved slice acquisition.

### 2.7 Data analysis

We used an event-related design for all of the analyses (i.e., to examine the effects of both difficulty and task), even though the various tasks were presented in mini-blocks. Only accurate responses were used in the analysis. All first-level and higher-level analyses were run using FMRI Expert Analysis Tool (FEAT) Version 5.98, in FMRIB's Software Library (FSL), www.fmrib.ox.ac.uk/fsl). Prior to inferential statistical analysis the following pre-processing was applied: Individual brain extraction (BET) to remove non-brain material from images for co-registration of the functional data, MCFLIRT motion correction (using fMRIB's Linear Registration Tool; Jenkinson et al., 2002), slice timing correction using Fourier-space time-series phase shifting (Sinc interpolation with a Hanning-windowing kernel), FWHM 6.0 mm spatial smoothing (Gaussian Kernel), high-pass temporal filtering (Gaussian-weighted least-squares straight line fitting, with sigma=100 s). We used FILM nonparametric estimation of time series autocorrelation (FILM; FMRIB's Improved Linear Model) to fit the model to the data, on all lower-level analyses. FSL's canonical gamma HRF along with a temporal derivative was used to model the HRF response. The first two volumes were removed to allow for T1 saturation effects. To analyse the data at the group level, we entered lower level FEAT directories into a higher level FMRIB'S Local Analysis of Mixed Effects (FLAME) Bayesian mixed effects analysis (Beckmann et al., 2003; Woolrich, 2008; Woolrich et al., 2004). *Z* (Gaussianised T/F) statistic images were thresholded using clusters determined by *Z*>2.3 and a (corrected) cluster significance threshold of *p*<.05 (Worsley, 2001). Names of brain areas reported are labelled according to the Harvard-Oxford Cortical Structural Atlas, Talairach Deamon and the Juelich Histological Atlas built into the FSLView software library.

Each task and condition was modelled separately using event based explanatory variables (EV) which were convolved to the haemodynamic response function (gamma function). We used a variable-epoch model as recommended by Grinband et al. (2008) to capture effects of time-on-task within each EV: the haemodynamic response function was aligned to the beginning of each correct trial and lasted for the duration of the event. Incorrect/removed trials were modelled as a separate EV, therefore, any data not modelled was included as rest. Several contrasts were run (11 in total): A contrast against rest/baseline was conducted for each of the six conditions (non-semantic easy, non-semantic hard, semantic verbal easy, semantic verbal hard, semantic picture easy, semantic picture hard), the hard version of each judgement type was contrasted against the corresponding easy version (non-semantic hard–non-semantic easy, semantic verbal hard–semantic verbal easy, etc.), and two contrasts examining modality/task were included (semantic verbal-rhyme; semantic picture–semantic verbal).

Finally, to test for differences in the effect of difficulty for verbal and picture semantic judgements across the whole brain, we computed the two-way interaction term between modality and difficulty at the higher level (in a model that also included main effects of difficulty and modality).

### 2.8 Region of interest analyses

To further investigate the contribution of brain regions implicated in semantic control by previous investigations (employing almost exclusively verbal stimuli), non-overlapping 8mm spherical ROIs were placed around peaks from the literature, guided by a recent meta-analysis of executive-semantic processing (Noonan et al., 2013b). This revealed a large swathe of activation across LIFG, plus peaks in RIFG pMTG and dAG (bordering IPS) but did not provide a clear means of examining different regions within IFG. Therefore, separate ROIs within BA44, 45 and 47 in LIFG were taken from an earlier fMRI study by Whitney et al. (2011a), which revealed increased activity to semantic control demands within these three distinct subregions. We transposed these spheres into the right hemisphere to investigate activation within RIFG. The left hemisphere pMTG and dAG coordinates were taken directly from the meta-analysis of Noonan et al. (2013b), using the contrast of more controlled vs. more automatic types of semantic processing. We also examined a site in ventral AG (vAG) linked to semantic processing but *not* executive control over semantic cognition. This was taken from the meta-analysis of Noonan et al. (2013b) from the contrast of all semantic>phonological tasks. The selection of ROIs was completely independent of the fMRI data to be analysed. ROI locations are shown in Table 6. The featquery tool in FSL was used to extract unthresholded mean parameter estimates for percent signal change for all of the voxels within each ROI, for each task over rest. These values were then subjected to ANOVA to establish the main effects of modality (verbal vs. picture semantics), task domain (phonology vs. verbal semantics) and difficulty, plus any interactions.

## 3 Results

### 3.1 Behavioural analysis

Table 4 shows behavioural data collected within the scanner. Analysis of these data revealed that the trials entered into the fMRI analysis were well-matched for RT. A 2×2 repeated-measures ANOVA examining modality (verbal vs. picture semantic judgements) and difficulty (strong vs. weak associations) revealed no effect of modality (*F*(1,21)=2.23, *p*=.15), a substantial effect of difficulty (*F*(1,21)=832.2, *p*<.001) and no interaction (*F*(1,21)<1, *p*=.47). Similarly, a 2×2 repeated-measures ANOVA comparing easy and difficult non-semantic and verbal semantic judgements found no main effect of task (*F*(1,21)=2.30, *p*=.14), a highly significant effect of difficulty (*F*(1,21)=1059.23, *p*<.001) and no interaction (*F*(1,21)=1.02, *p*=.32).

### 3.2 Whole brain analyses

Whole brain analyses of activation against rest for each type of task (e.g., verbal semantic, picture semantic, easy non-semantic rhyme task and harder non-semantic segmentation task) showed activity for all tasks in left anterior and posterior IFG (BA 44, 45, 47) extending into left premotor cortex, right posterior IFG, left and right IPS/IPL and visual cortex (see Supplementary Figs. S1 and S2). There was recruitment in left and right STG for the non-semantic language tasks and verbal semantic tasks. This response extended into superior parts of the left anterior temporal lobe and left anterior IFG for the verbal semantic task. We did not observe activation of ATL for the picture semantic task, despite evidence that this brain region supports multimodal semantic representations, possibly because picture-based tasks elicit peak activation in anterior fusiform cortex; this ventromedial part of ATL is affected by signal loss and distortion in gradient echo EPI (Visser et al., 2009, 2012). The harder non-semantic task (involving segmentation) showed additional areas of recruitment in prefrontal cortex, particularly within inferior frontal sulcus bilaterally, reflecting the strong executive demands of these judgements.

**Fig. 1:**
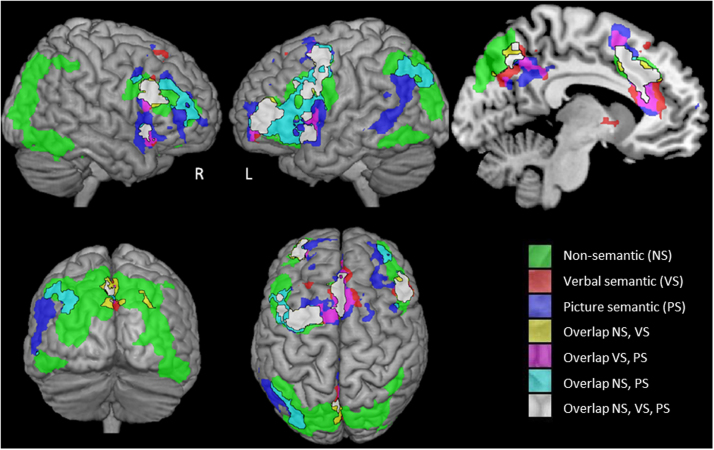
Whole brain analysis contrasts of hard over easy for all three tasks (cluster correction, *Z*≥2.3, *p*<.05). L=left, R=right hemisphere. NS=non-semantic tasks (easy=rhyme judgement; hard=problem solving task involving segmentation). VS=verbal semantic. PS=picture semantic.

Contrasts between easy and hard versions of each task were used to investigate which areas showed differential activity when task demands were increased. These contrasts can be found in Table 5 and Fig. 1: only clusters showing significant activation after cluster correction (*Z*=2.3, *p*<.05) are reported. Sites responding to difficulty across tasks and modalities (shown in white in Fig. 1) were found in left posterior IFG extending into inferior frontal junction (IFJ) and premotor areas, right posterior IFG/IFJ, frontal operculum bilaterally, a site within medial PFC (pre-SMA plus regions within anterior cingulate; ACC) and precuneus: these regions have been shown to co-activate under conditions of high executive demands and correspond to key parts of the ‘multi-demand’ or ‘dorsal attention system’ network (Duncan, 2010; Woolgar et al., 2011). The contrast between the easy (rhyme) and executively-demanding (segmentation) phonological tasks (in green in Fig. 1) revealed additional responses within this multi-demand network: namely, within bilateral IFS, bilateral IPS and bilateral temporal–occipital cortex. Several of these regions were also activated by the contrast of easy and difficult picture semantic judgements (in cyan), confirming their involvement in demanding tasks across input modalities and judgement types. There were relatively few regions specifically recruited in the contrast of easy and difficult verbal semantic judgements that were not also activated by the contrast of easy and harder non-semantic tasks: this pattern was seen most notably in a ventral extension of the pre-SMA/ACC activity and in a small region within anterior LIFG (shown in red or pink in Fig. 1). Although no sites within temporoparietal cortex were implicated in demanding decisions across verbal and picture semantic tasks, left pMTG extending into the angular gyrus was revealed in the contrast of hard as opposed to easy picture semantic decisions, supporting the view that pMTG co-activates with LIFG in executively-demanding semantic tasks (Noonan et al., 2013b; Whitney et al., 2011a, 2011b). There were also IFG sites which showed a response modulated by difficulty in the picture semantic task only, particularly in the right hemisphere.

As noted above, these overlays of the effect of difficulty in each task suggest a common response to control demands across tasks and modalities in large swathes of LIFG and left IFS, left IFJ, right posterior IFG, right and left frontal operculum, plus pre-SMA/ACC, precuneus and perhaps IPS. However, it is unclear from this analysis whether there were any brain regions that showed a greater response to difficulty for verbal or picture semantic judgements. To address this issue, we computed a 2×2 ANOVA, examining modality (semantic judgements to words vs. pictures), difficulty and the interaction of these factors. There were clear effects of modality, some of which were within brain regions that showed a stronger response to more difficult judgements (see Supplementary materials, Fig. S3). However, the interaction between difficulty and modality did not reveal any significant clusters that could be interpreted as a greater response to difficulty (e.g., hard>easy trials) for a particular modality (see Supplementary materials, Fig. S4). The interactions that were seen reflected instead a greater response to *easy* trials in particular modalities.

### 3.3 Region of Interest Analyses (ROI)

**Fig. 2:**
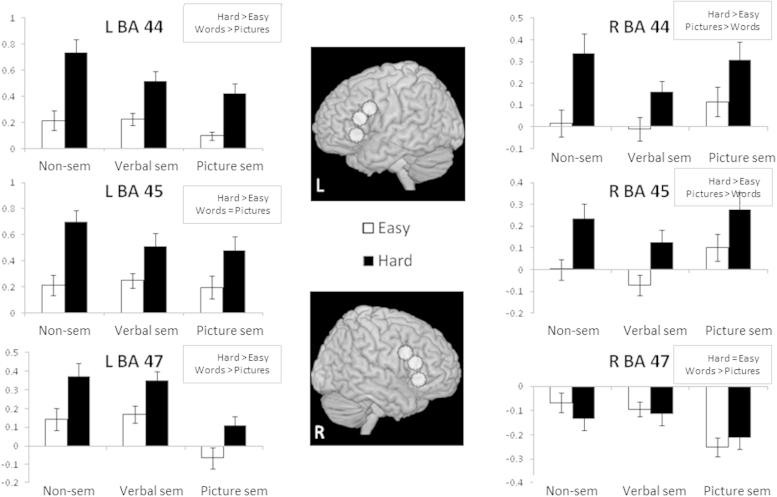
ROI analyses of percentage signal change in left and right IFG (8 mm spheres centred around peaks in Brodmann area 44, 45 and 47 from Whitney et al. (2011a) and implicated in semantic control). Error bars indicate standard error of the mean. L=left hemisphere; R=right hemisphere. The charts show easy and hard judgements for non-semantic tasks (non-sem), verbal semantics (verbal sem) and picture semantics (picture sem). Significant effects are summarised next to each graph (see Table 6 for details).

**Fig. 3:**
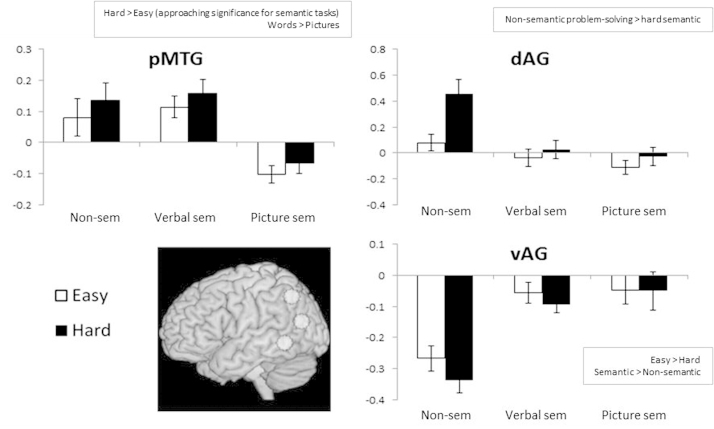
ROI analyses of percentage signal change in left temporoparietal cortex; pMTG=posterior middle temporal gyrus, dAG=dorsal angular gyrus, vAG=ventral angular gyrus; 8 mm spheres centred around peaks from Noonan et al. (2013b). Error bars indicate standard error of the mean. The charts show easy and hard judgements for non-semantic tasks (non-sem), verbal semantics (verbal sem) and picture semantics (picture sem). Significant effects are summarised next to each graph (see Table 6 for details).

Since our aim was to establish how the semantic control network previously established for verbal tasks (e.g., by the meta-analysis of Noonan et al., 2013b) might be modulated by modality, we examined possible interactions between difficulty and modality or task in the following ROIs that were highlighted by that review of the literature: posterior, mid and anterior IFG in the left and right hemispheres and left pMTG, dAG, and vAG. Percentage signal change values were extracted for each individual participant for each condition. We computed two 2×2 ANOVAs: the first examined the main effects of task (verbal semantic vs. non-semantic), difficulty within those tasks and their interaction, while the second examined the main effects of modality (verbal semantic vs. picture semantic) and difficulty of these semantic tasks plus their interaction. These ANOVAs are reported in Table 6 and the data are summarised in Figs. 2 and 3.

The left inferior frontal gyrus ROIs showed a significant effect of difficulty across all three tasks (*F*(1,21)>15.48, *p*≤.001; Table 6). For left BA 44 and BA 45, a direct comparison of the non-semantic and verbal semantic tasks revealed a greater effect of difficulty for the non-semantic than the verbal semantic task at both sites (*F* (1,21)>5.16, *p*≤.03), presumably reflecting the considerable executive demands and problem-solving requirements of the hard non-semantic task. Left BA 44 also showed more activity for verbal than picture semantic judgements (*F*(1,21)=8.2, *p*=.009), while the response of BA 45 to semantic tasks was not modulated by modality (F(1,21)<1, *p*=.52). Left BA 47 showed a distinct pattern: unlike posterior parts of LIFG, this site did *not* show a stronger effect of difficulty for the non-semantic than the verbal semantic task (F(1,21)<1, *p*=.55); yet there was a greater response for verbal than picture semantic judgements (*p*<.001). All three sites in LIFG showed an equivalent effect of difficulty for the picture and word semantic tasks (interaction of modality and difficulty: *F*(1,21),<1, *p*>.65).

Right BA 44 and 45 also responded to the difficulty manipulation across all three tasks (*F*(1,21)>21.2, *p*<.001), however right BA 47 did not show increased activation for difficult decisions, in contrast to all other IFG sites (*F*(1,21)<1.3, *p*≥.26). Right BA 44 and BA 45 showed greater recruitment for the non-verbal than the verbal semantic task (main effect of task, *F*(1,21)>5.03, *p*≤.036), but in contrast to LIFG, there was greater activation for the picture than the verbal semantic modality (*F*(1,21)>10.5, *p*≤.004). Again, right BA 47 showed a different pattern: there was no significant difference between the non-semantic and verbal semantic task at this site (*F*(1,21),<1, *p*>.95), but there was greater deactivation for the pictures than verbal judgements (i.e., a reversal of the modality effect observed in right BA 44/45; words>pictures, *F*(1,21)=9.06, *p*=.007).

ROI results for posterior temporal and inferior parietal sites in the left hemisphere are shown in Fig. 3. In pMTG, there was a greater response to verbal than to picture semantic tasks (*F*(1,21)=42.0, *p*<.001); indeed the picture task showed deactivation relative to rest. There was also a near-significant effect of difficulty for the semantic tasks (*F*(1,21)=3.28, *p*=0.08), which did not interact with modality. pMTG did not show a stronger response to the executively-demanding non-semantic task compared with the verbal semantic task, or a larger effect of difficulty for the non-semantic than the verbal semantic task, consistent with previous findings that pMTG makes a relatively specific contribution to semantic control (Noonan et al., 2013b). In dAG (implicated in semantic control by Noonan et al., 2013b), there was a substantial difference between non-semantic and verbal semantic tasks (*F*(1,21)=25.5, *p*<.001), with percentage signal change near zero for all of the tasks except the demanding non-semantic condition (which had significant problem-solving requirements). As a result, the comparison of non-semantic and verbal semantic tasks revealed a main effect of difficulty (*F*(1,21)=25.7, *p*<.001) and an interaction between task and difficulty (*F*(1,21)=19.1, *p*<.001). This site showed no difference between the verbal and picture semantic tasks (*F*(1,21)=2.79, *p*=.11) and an effect of difficulty for semantic tasks that approached significance (*F*(1,21)=3.93, *p*=.06).

vAG (implicated in more automatic aspects of semantic processing by Noonan et al., 2013b) unsurprisingly showed a different pattern from the other sites: there was deactivation for all tasks relative to rest, and this was greater for more difficult decisions. There was more deactivation for non-semantic than verbal semantic judgements; *F*(1,21)=39.9, *p*<.001, consistent with previous reports of effects of semantic contrasts at this site). There was also a reverse effect of difficulty (more deactivation for difficult decisions; *F*(1,21)=9.73, *p*=.005). vAG showed equivalent deactivation across the verbal and picture semantic tasks (*F*(1,21)<1, *p* =.60), and no effect of difficulty for semantic judgements (*F*(1,21)<1, *p*=.341).

## 4 Discussion

This study examined the brain’s response to relatively automatic and more controlled forms of semantic retrieval from different modalities (words and pictures) to establish whether sites previously implicated in semantic control within left and right IFG, pMTG and dorsal AG show an *amodal* response. Previous investigations have almost exclusively used verbal stimuli and have focussed on the role of LIFG (see Noonan et al., 2013b) yet semantic aphasia patients have difficulty with controlled semantic retrieval in both verbal and non-verbal tasks after stroke affecting LIFG and/or left temporoparietal cortex (e.g., words, pictures, sounds, objects; Corbett et al., 2009a, 2009b; Gardner et al., 2012; Jefferies and Lambon Ralph, 2006; Noonan et al., 2010). Thus the data we provide are novel and important in advancing our understanding from neuropsychology. We also compared relatively easy non-semantic decisions (based on rhyme relationships) with more difficult non-semantic judgements (requiring explicit segmentation, manipulation and matching of phonemes), since this contrast reveals which sites implicated in semantic control also show a response to non-semantic executive demands.

The results showed (1) graded modality effects across the hemispheres in posterior IFG (pIFG): left pIFG made a greater contribution to challenging verbal semantic decisions, while right pIFG responded more to picture-based decisions. (2) There was a second anterior-to-posterior functional gradient within left IFG, with posterior regions making a greater contribution to *non-semantic* control, and anterior LIFG (aIFG) showing a selective response to *verbal semantic* tasks. (3) The only ROI to show a truly multimodal response to controlled retrieval demands was in left mid-LIFG (BA45). (4) The analyses also provided some additional evidence for a distributed network underpinning semantic control across modalities. pMTG showed a near-significant effect of difficulty for semantic tasks, plus a stronger overall response for the verbal than picture semantic judgements. (5) Although dAG/IPS has been previously implicated in semantic (and domain general) control (Noonan et al., 2013b), the response in this study was largely restricted to the hard non-semantic judgements which maximised executive demands. We also confirmed the strong functional dissociation between dorsal and ventral AG, with the latter showing reverse difficulty effects and task-related deactivation.

### 4.1 Inferior frontal gyrus (IFG)

The functional specialisation of left IFG by language task (phonology vs. semantics) has received attention in previous studies (e.g., Devlin et al., 2003; Gold and Buckner, 2002; Gough et al., 2005; Nixon et al., 2004); however, few if any studies have manipulated semantic input modality (words vs. pictures) and difficulty simultaneously. Broadly, our findings confirm previous literature showing involvement of LIFG in verbal control (Badre et al., 2005; Thompson-Schill et al., 1997; Whitney et al., 2009, 2011b), while uniquely revealing that this region also contributes to *semantic control for non-verbal stimuli*. In particular, a region within left mid-LIFG (BA45) showed a greater BOLD response to more difficult judgements across all tasks and modalities. Left BA45 responds reliably across many different manipulations of semantic control (Noonan et al., 2013b) and its activation is driven by both controlled retrieval demands (higher when the probe-target relationship is weak) and competition/selection (for example, when there are more competitors or strong distracters) (Badre et al., 2005; Nagel et al., 2008; Thompson-Schill et al., 1997). BA45 lies adjacent to inferior frontal sulcus (IFS), a site in the multi-demand cognitive control network which shows an increased response to more demanding tasks across all cognitive domains (Duncan, 2010; Duncan and Owen, 2000). The activation linked to difficulty across tasks in our whole-brain contrasts extended into IFS, as expected. However, Nagel et al. (2008) disambiguated semantic selection from response selection, and found that both BA 45 and 47 responded exclusively to *semantic* selection, while dorsolateral prefrontal cortex (BA 9/46) was recruited for *response* selection. Therefore, although BA45 makes a broad contribution to semantic and linguistic control across tasks and modalities, it may not qualify in full as a multiple demand region: instead, its contribution appears to be more restricted to the control of *non-spatial* attention within the ventral stream (Blank et al., 2014; Fedorenko et al., 2012; Nobre et al., 2004).

Other parts of IFG showed a degree of functional specialisation. There was graded *hemispheric specialisation in posterior IFG* across tasks/modalities: left BA44 showed a stronger response to phonological than verbal semantic tasks, while right BA44 showed a stronger response to conceptual tasks employing pictures (cf. Adams and Janata, 2002; Kelley et al., 1998). In right BA 44 and 45, the response to the easy phonological and verbal semantic tasks was not above baseline, yet these sites responded significantly to the more challenging verbal judgements. This confirms that *semantic and linguistic control processes are bilateral* in line with our recent ALE meta-analysis of neuroimaging studies of semantic control (Noonan et al., 2013b). The graded effect of modality across left and right posterior IFG may reflect differential connectivity with left and right posterior temporal areas that show some degree of specialisation for words and pictures, and/or a division of labour between the hemispheres in their contribution to semantic and cognitive control.

There was also graded specialisation within LIFG from *anterior to posterior* areas. Left posterior IFG (BA 44) showed a greater overall response to verbal than picture semantic judgements and a greater increase in activity for hard non-semantic than verbal semantic decisions. These findings are consistent with studies that particularly implicate this region in control over phonology (Blumstein et al., 2005; Nixon et al., 2004; Ojemann and Mateer, 1979; Poldrack et al., 2001; Poldrack et al., 1999; Romero et al., 2006) and with recent neuroimaging meta-analyses that report activity in posterior LIFG for both phonology and semantics (Noonan et al., 2013b; Vigneau et al., 2006). However, the hard non-semantic task also strongly loaded problem-solving and executive aspects of working memory, so we cannot be certain if the recruitment of posterior LIFG in this task was linked to phonological processing per se.

In contrast, anterior LIFG (BA 47) did not show this greater response to the non-semantic tasks, in line with previous studies that found relative specialisation for semantic processing in this region (Badre et al., 2005; Devlin et al., 2003; Gough et al., 2005; Wagner et al., 2001). Posterior LIFG (BA44) may contribute to the control of language stimuli irrespective of semantic demands because it has strong connections with language-sensitive STG via the arcuate fasciculus (Anwander et al., 2007; Petrides and Pandya, 2002), and weaker structural and functional connections to multimodal temporal lobe sites involved in semantic processing (Friederici, 2009; Xiang et al., 2010). In contrast, mid and anterior LIFG (BA 45 and 47) are more strongly connected to multimodal semantic areas such as ATL, via the extreme capsule (EmC), plus pMTG and inferior temporal lobe areas via the inferior longitudinal fasiculus (Anwander et al., 2007; Xiang et al., 2010).

Hemispheric differences were strikingly different between anterior and posterior IFG. While pIFG showed clear hemispheric specialisation based on input modality (see above), this pattern was not seen in aIFG. Right aIFG showed task-related deactivation, which was more pronounced for picture than word tasks. Therefore, direct contrasts of words and pictures in left and right aIFG showed the same pattern: a positive signal change to words. Badre et al. (2005) implicated aIFG in “controlled retrieval” and this process might be maximised during the identification of weak semantic associations for words, since picture stimuli include additional information about linking context. For example, in the “duck” with “gun” example (Table 1), the picture shows a *shotgun*, and this additional information (not available from the word”gun” alone) helps to establish that a’hunting’ context links the probe and target, reducing the controlled retrieval demands. Similarly, BA 47 activity has previously been reported to be stronger for abstract/concrete judgements (in comparison to living/non-living decisions) for both pictures and words (Wagner et al., 1997), suggesting that this site may be modulated by the ‘abstractness’ of a decision as opposed to modality per se. This proposal is related to the hypothesis that anterior parts of PFC contribute to abstract/complex forms of control, such as identifying which features of a concept should be in the focus of attention (Badre and D’Esposito, 2009). In contrast, posterior parts of IFG may contribute to the selection of these features (i.e., channelling activation towards gun features relevant for *hunting* as opposed to *gangland violence*), once abstract priorities for processing have been established in aIFG.

### 4.2 Left posterior Middle Temporal Gyrus (pMTG)

Both neuropsychology and neuroimaging studies predict dissociations within left temporoparietal cortex between sites that are expected to contribute to semantic control (i.e., pMTG; dAG/IPS) and those that are implicated in semantic processing but do not show effects of control requirements (vAG). Posterior portions of MTG show strong anatomical and functional connections with LIFG (Catani et al., 2005; Saur et al., 2008; Turken and Dronkers, 2011; Xiang et al., 2010), suggesting that these two cortical regions might work together to control the retrieval of conceptual representations such that semantic processing is appropriate for a given task or context. SA patients – who show deregulated semantic cognition across both verbal and non-verbal tasks – have damage to LIFG and/or left posterior temporal cortex encompassing pMTG, and patients with anterior and posterior lesions show comparable deficits on many semantic tasks (Corbett et al., 2011; Gardner et al., 2012; Jefferies et al., 2007; Noonan et al., 2010). Moreover, convergent evidence for a role of left pMTG in executive-semantic processing is provided by TMS (Whitney et al., 2011b) and a neuroimaging meta-analysis found reliable activation of this site in response to a wide variety of manipulations of semantic control demands (Noonan et al., 2013b). Consistent with these studies, we found some evidence of an effect of difficulty within left pMTG. First, the whole brain contrasts revealed stronger recruitment for harder than easier *picture* semantic judgements in ventral occipital-temporal cortex, extending into pMTG. This effect partially overlapped with the ventral posterior temporal site showing an effect of modality (pictures>words) and difficulty (hard>easy) in Figure S1. Our ROI, taken from the Noonan et al. (2013b) meta-analysis, was focussed on MTG not ITG and showed a near-significant effect of difficulty for semantic judgements and a stronger response to words than pictures at this location.

Overall, our findings are consistent with a recent theoretical perspective which suggests that LIFG and pMTG form a large-scale distributed network that focuses semantic processing on currently-relevant aspects of knowledge in a flexible way according to the current context (Noonan et al., 2013b). Turken and Dronkers (2011) suggested that interactions between LIFG and pMTG might allow task-relevant aspects of meaning to be sustained in STM so that they can be integrated into the overall sentence (or linking context). While these authors were specifically focussed on verbal processing, pMTG is involved in comprehending pictures as well as words: this site is revealed by a conjunction of verbal and non-verbal semantic tasks in fMRI, and TMS to this region disrupts both picture and word semantic associations (Chee et al., 2000; Hoffman et al., 2012; Vandenberghe et al., 1996; Visser et al., 2012). We therefore suggest that a flexible form of contextual integration is necessary for understanding the significance of objects encountered non-verbally, as well as through words, especially when processing items that are not highly associated.

However, this account does not predict the striking effects of modality that we also observed within pMTG in our ROI analysis: there was task-related activation for the verbal semantic task yet *deactivation* for the picture task. At first glance, this aspect of our data is consistent with an influential account of the role of pMTG in speech processing, which suggests that this region maps between lexical representations and concepts (Hickok and Poeppel, 2000, 2007). However, as noted above, adjacent regions in pMTG are involved in comprehending pictures as well as words. Different literatures on speech comprehension and conceptual processing have therefore reached apparently contradictory conclusions about the contribution of pMTG to non-verbal semantic tasks. The complex responses in the pMTG ROI – namely an effect of control demands and task-positive responses to spoken words yet task-negative responses to pictures – resembles the pattern observed in aIFG and could be explained in a similar way: i.e., the co-activation of aIFG and pMTG may allow semantic features relevant to the linking context to be brought to the fore (e.g., the context of *hunting* in the “duck” and “gun” example). This requirement is tapped by weak picture associations to a degree but the demands are greater for weak verbal associations, since pictures provide additional cues to the linking context. This interpretation is also broadly consistent with Noonan et al.'s (2013b) finding that left aIFG and left pMTG are implicated in control for semantic tasks more than other language tasks, since the requirement to identify and maintain features relevant to the current context is a particular requirement of semantic judgements.

### 4.3 Angular Gyrus (AG)

Angular gyrus has often been implicated in semantic cognition, and recent evidence suggests that there are several functional specialisations within this large area (Noonan et al., 2013b; Seghier et al., 2010). Dorsal aspects of AG bordering IPS are involved in several non-semantic domains (e.g., reading, visuospatial search and ‘number line’ tasks, left/right discrimination; Carreiras et al., 2009; Gobel et al., 2001; Hirnstein et al., 2011), as well as in semantic judgements (e.g., Noonan et al., 2013b; Price, 2010; Seghier et al., 2004; Sharp et al., 2010; Vigneau et al., 2006), and this site forms part of the frontoparietal ‘multi-demand’ control system (Duncan, 2010; Duncan and Owen, 2000). Our ROI analysis of dAG found a significant contribution of this site to the hard non-semantic task involving phonological segmentation but only a marginal effect of difficulty for the semantic tasks, even though dAG was found to be a key site responding to the control demands of semantic tasks in the meta-analysis of Noonan et al. (2013b). One possible explanation for this pattern is provided by previous fMRI and TMS studies which reported that dAG/IPS plays a critical role in *goal-driven* aspects of semantic control, such as retrieving a particular semantic feature in line with task instructions, but not in *stimulus-driven* aspects of control, when the nature of the stimuli themselves and not the task instructions determine the executive demands (Badre et al., 2005; Whitney et al., 2012). In the experiment reported here, difficult judgements required controlled retrieval because the probe and target concept were distantly associated, not because participants were specifically instructed to retrieve non-dominant aspects of semantic information. Therefore, a working hypothesis is that, for weakly-linked items such as “duck-gun”, interactive processing within LIFG and pMTG is important for uncovering a context (hunting) that can be used to link these words and for focussing semantic processing on features of the concepts that are relevant to the context. In contrast, when the task instructions require participants to focus on pre-specified non-dominant aspects of knowledge (e.g., do two concepts share a colour?), dAG/IPS may additionally be engaged in the flexible and strategic allocation of attention to the properties of the stimulus which are relevant to the task. The contrast between easy and hard non-semantic decisions, which elicited the most substantial activation within dAG/IPS, maximised this requirement for goal-driven attention: in the hard task, participants were instructed to perform phoneme matching on either the first or last phoneme, while in the easy task, target rhyming words could be detected on the basis of the stimuli themselves and without explicit instructions.

Ventral angular gyrus (vAG) showed a different pattern: there was task-related deactivation, relative to rest, for all of the tasks. There was no increase in the response of this region with difficulty: instead, more difficult tasks elicited more *deactivation* and as a consequence this site showed a reverse effect of difficulty (i.e., easy>hard decisions; the opposite pattern to IFG, pMTG and dAG/IPS; cf. McKiernan et al., 2003). In addition, there was greater deactivation in vAG for phonological than semantic tasks. This finding is consistent with neuroimaging meta-analyses, which have observed strong responses within vAG when semantic tasks are compared with non-semantic tasks (Humphreys and Lambon Ralph, 2014; Noonan et al., 2013b), even when these tasks are matched for difficulty/RT (Binder et al., 2009), yet no response in a contrast of high control over low control semantic tasks (Noonan et al., 2013b). These findings suggest that vAG is involved in relatively automatic aspects of semantic processing – consistent with the finding of activity in this region even when the demands on executive control are minimised, for example in naturalistic comprehension tasks (Humphreys and Lambon Ralph, 2014; Spitsyna et al., 2006). Moreover, a recent investigation of angular gyrus suggested that while dAG is involved in the search for an appropriate semantic representation, the role of vAG is likely to be in conceptual identification, as this site showed activation for meaningful items, but deactivation for meaningless items (Seghier et al., 2010).

In summary, this study manipulated difficulty, task domain (semantic vs. non-semantic judgements) and semantic input modality (decisions to words and pictures), to explore differentiation of function within the executive-semantic network. We found that while left mid-IFG (BA45) responded to executive control demands irrespective of task and modality, other parts of IFG showed graded specialisation across modalities and tasks. In particular, posterior IFG, implicated in the selection of semantic and non-semantic information, showed specialisation across the hemispheres according to modality. Moreover, in posterior cortical regions, we observed a dissociation between (i) regions showing task-related deactivation and reverse difficulty effects – indicative of automatic aspects of semantic retrieval (ventral AG), and (ii) regions showing task-positive activation with stronger signal for more difficult conditions, in sites linked to more controlled aspects of semantic retrieval (pMTG; dAG/IPS). The similarity of the response in left aIFG and pMTG is consistent with the view that co-activation of these regions is important for shaping semantic processing such that it is focussed on the currently-relevant semantic context which supplies a link between weakly associated items (e.g., the hunting context for duck and gun). This process is more strongly taxed by word than picture association judgements, since the concrete features of pictures provide additional cues to the linking context.

## Figures and Tables

**Table 1:** Task conditions. Words in quotation marks were presented as auditory probes and did not appear on the screen. The non-semantic easy task involved rhyme judgement, while the non-semantic hard task involved phoneme segmentation – in this example, participants were instructed to strip the final sound from the probe and use it to create a word from one of the possible targets. The semantic tasks involved association matching, with either words or pictures. Easy trials involved strong associations, while hard trials involved weaker associations.

**Table 2:** Rated strength of association for the verbal semantic and picture semantic stimuli included in the fMRI analysis. Table shows means and standard deviations (in parentheses) on a scale from 1 to 5.

Modality	Easy (strong associations)	Hard (weak associations)
Verbal semantic	1.60 (.11)	2.08 (.09)
Picture semantic	1.57 (.08)	2.11 (.12)

**Table 3:** Psycholinguistic properties of the stimuli included in the fMRI analysis. Table shows means and standard deviations (in parentheses) for word frequency (counts per million) and imageability.

Task	Celex written		Imageability	
Easy	Hard	Easy	Hard

*Probes*				
Non-semantic	3.96 (.37)	3.84 (.36)	600 (11)	602 (11)
Verbal semantic	3.95 (.42)	3.97 (.45)	598 (16)	592 (15)
Picture semantic	3.83 (.35)	3.95 (.42)	598 (22)	591 (19)
				
*Targets*				
Non-semantic	4.03 (.20)	3.92 (.22)	558 (10)	564 (14)
Verbal semantic	4.09 (.25)	4.04 (.28)	599 (8)	589 (10)
Picture semantic	4.03 (.30)	4.27 (.23)	594 (13)	592 (7)

**Table 4:** RT on correct trials in seconds (standard error in parentheses) from 22 participants entered into the event-related fMRI analysis.

	Easy (s)	Hard (s)
Non-semantic	1.3 (.06)	2.6 (.08)
Verbal semantic	1.34 (.03)	2.71 (.07)
Picture semantic	1.29 (.04)	2.6 (.09)

**Table 5:** List of sites emerging from hard>easy contrasts. Anatomical labels were provided by the Harvard-Oxford Atlas implemented in FSL.

Brain region	Phonological Hard>Easy							Verbal semantic hard>Easy							Picture semantic hard>Easy						
Activation peaks	BA	*Z*	*x*	*y*	*z*	Voxels	Peaks	BA	*Z*	*x*	*y*	*z*	Voxels	Peaks	BA	*Z*	*x*	*y*	*z*	Voxels
Cingulate Gyrus/Medial Frontal	L PAC	32	5.53	−6	26	36	8307	L PAC	32	4.6	−4	28	32	4412	L MFG^‡^	10	5.14	−30	50	4	13,938
L PAC	32	5.46	−2	22	38		L PAC	32	4.59	−4	26	36		R PAC/Med FG	32	4.88	4	34	28	
L MFG/PMC	~6	5.32	−28	0	46		L PAC/SFG		4.5	0	18	50		R PAC	32	4.86	2	34	32	
L IFG	44/45	4.92	−48	22	20		R SFG	8	4.43	4	24	52		R PAC/SFG	32/8	4.82	0	20	48	
L PrecG/IFG	44	4.91	−40	6	26		R SFG	8	3.91	0	30	46		R SFG‡	10	4.78	32	50	8	
L MFG/PrecG/IFG	9/44	4.75	−44	8	32		L CGa	24	3.91	−6	28	22		L IFG	44	4.52	−52	14	10	
																				
Parietal Lobe	L SPL	7	5.61	−26	−70	34	13,406	R SPL/PCN		3.49	2	−60	50	1504	L CG/PCN	31	3.6	−4	−48	40	925
R SPL/OL	7	5.52	28	−70	40		R PL/PCN	~31	3.49	16	−62	24		L PCN		3.49	−6	−64	44	
R PL‡		5.42	28	−70	32		L PCN/SPL	7	3.44	−8	−68	34		L PCN		3.43	−4	−54	42	
L AG	40	5.15	−36	−58	40		L PCN/SPL	7	3.43	−4	−70	34		L CG/PCN	31	3.4	−12	−50	34	
L SPL/PCN	7	5.09	−24	−76	44		L PCN/SPL	7	3.42	−6	−64	50		L PCN		3.23	−12	−64	26	
L SPL/PCN	7	5.01	−16	−74	48		L PCN/SPL	7	3.32	−6	−58	48		L PCN		3.06	−4	−68	38	
																				
Temporal Gyri	L TOF/FFG		4.44	−30	−52	−10	1309	L IFG/L aTG	47/38	4.25	−34	22	−10	957	L AG	39	4.47	−56	−60	26	1976
L pITG	37/20	4.36	−52	−58	−12		L aSTG/L IFG	38/47	3.46	−46	14	−10		L pMTG		3.77	−56	−44	6	
L pITG	37	4.32	−52	−62	−14		L Pallidium		3.45	−14	0	−4		OL		3.68	−34	−80	40	
L TOF/FFG	37	4.07	−24	−54	−10		L aSTG/L IFG	38/47	3.39	−52	20	−12		L pMTG		3.67	−62	−40	−4	
L pITG	37	3.5	−52	−42	−14		L IFG	44/45	3.22	−48	20	2		L pMTG		3.42	−58	−40	−8	
L OFG	18	2.77	−28	−72	−14		L caud/put		3.02	−16	18	−6		L pSMG	40	3.22	−62	−50	34	
																				
Frontal Gyri	R INS		4.61	32	22	−6	2713	L MFG	10	4.45	−32	48	6	748							
	R MFG/IFG	9/44	4.2	50	22	30		L SFG‡	10	3.23	−22	48	4								
	R PrecG/IFG	~9/44	4.18	42	6	28		L SFG	10	3.14	−26	62	−10								
	R IFG	45	4.14	46	32	12		L SFG	10	3.13	−28	62	−6								
	R FC	46	4.1	50	38	16		L MFG		3.04	−28	58	2								
	R FC‡	11	3.63	22	40	−16		L Med FG	11	2.87	−16	58	−14								
														697							
								R Insula		3.9	32	20	−4								
								R IFG/INS	47	3.75	40	18	−12								
								R IFG	47	3.63	30	16	−18								
								R IFG/INS	47	3.22	42	20	−6								
								R FOC		3.13	22	8	−24								
								R FOC		2.95	22	8	−20								
														565							
								R IFG	44/45	4.08	54	24	18								
								R IFG	44	3.66	54	22	26								
								R MFG	9	3.4	48	26	30								
								R IFG	45	3.19	60	24	12								
								R IFG‡	~45	2.8	36	20	16								

*Notes*: MFG=middle frontal gyrus, PMC=premotor cortex, IFG=inferior frontal gyrus, PrecG=precentral gyrus, SPL=superior parietal lobule, AG=angular gyrus, pITG=posterior inferior temporal gyrus, OFG=Occipital Fusiform Gyrus, FFG=fusiform gyrus, SFG=superior frontal gyrus, TG=temporal gyrus, STG=superior temporal gyrus, pMTG=posterior middle temporal gyrus, SMG=supramarginal gyrus, TOF=temporal occipital fusiform cortex, PL=parietal lobe, FOC=frontal orbital cortex, PAC=Paracingulate Gyrus, FC=frontal cortex, PCN=precuneus, CGa=Anterior Cingulate Gyrus, CG=cingulate gyrus, caud=caudate, put=putamen, OL=lateral occipital cortex, INS=insular cortex, med=medial.

‡ Sub-gyral.

**Table 6:** *F* values for ANOVAs comparing percentage signal change in left (L) and right (R) IFG (BA 44, 45, 47), left pMTG (posterior middle temporal gyrus), left dAG (dorsal angular gyrus), and left vAG (ventral angular gyrus).

	ROI coordinates	*df*	Effect of task (phonology vs. verbal semantic)			Effect of modality (verbal vs. picture semantic)			
Task	Difficulty	Task×Difficulty	Modality	Difficulty	Modality×Difficulty	Notes
L BA 44	−48 16 28	1, 21	2.70	63.2⁎⁎⁎	8.86⁎⁎	8.2⁎⁎	29.6⁎⁎⁎	<1	**Words**>Pictures; Hard>Easy; Non-semantic problem-solving>Hard semantic
L BA 45	−48 28 16	1, 21	1.93	46.3⁎⁎⁎	5.16⁎	<1	25.0⁎⁎⁎	<1	**No modality effects**; Hard>Easy; Non-semantic problem-solving>Hard semantic
L BA 47	−48 32 −4	1, 21	<1	29.0⁎⁎⁎	<1	21.0⁎⁎⁎	15.5⁎⁎⁎	<1	**Words**>Pictures; Hard>Easy
R BA 44	48 16 28	1, 21	6.88⁎	33.2⁎⁎⁎	2.82	10.5⁎⁎	30.3⁎⁎⁎	<1	**Pictures**>Words; Hard>Easy; Non-semantic>Semantic
R BA 45	48 28 16	1, 21	5.03⁎	31.0⁎⁎⁎	<1	12.4⁎⁎	21.2⁎⁎⁎	<1	**Pictures**>Words; Hard>Easy; Non-semantic>Semantic
R BA 47	48 32 −4	1, 21	<1	1.33	<1	9.06⁎⁎,†	<1	<1	**Words**>Pictures (in deactivation); Hard=Easy
L pMTG	−55 −50 −5	1, 21	1.07	2.83	<1	42.0⁎⁎⁎	3.28°	<1	**Words**>Pictures; Hard>Easy (approaching significance)
L dAG	−41 −59 46	1, 21	25.5⁎⁎⁎	25.7⁎⁎⁎	19.1⁎⁎⁎	2.79	3.93°†	<1	Non-semantic problem-solving>Hard semantic
L vAG	−44 −69 19	1, 21	39.9⁎⁎⁎,†	9.73⁎⁎,†	<1	<1	<1	<1	Easy>Hard; Semantic>Non-semantic (in deactivation)

⁎⁎⁎ *p*≤.001.

⁎⁎ *p*≤.01.

⁎ *p*≤.05.

° ≤.1.

† Effects below zero (i.e., deactivation). ROI coordinates for IFG from Whitney et al. (2011a). ROI coordinates for pMTG, dAG and vAG from Noonan et al. (2013b). Coordinates are given in MNI space (converted from Talairach space for Noonan 2013 peaks using BrainMap GingerALE 2.3).

## Footnotes

Appendix A Supplementary data associated with this article can be found in the online version at doi:10.1016/j.neuropsychologia.2015.02.030.
