# Supplementary material for: Conceptual control across modalities: graded specialisation for pictures and words in inferior frontal and posterior temporal cortex
Source: Neuropsychologia. 2015 Sep;76:92–107. doi: 10.1016/j.neuropsychologia.2015.02.030 (PMC4582805; doi:10.1016/j.neuropsychologia.2015.02.030)
Supplement: Supplementary file 1 — Supplementary material [file mmc1.docx]

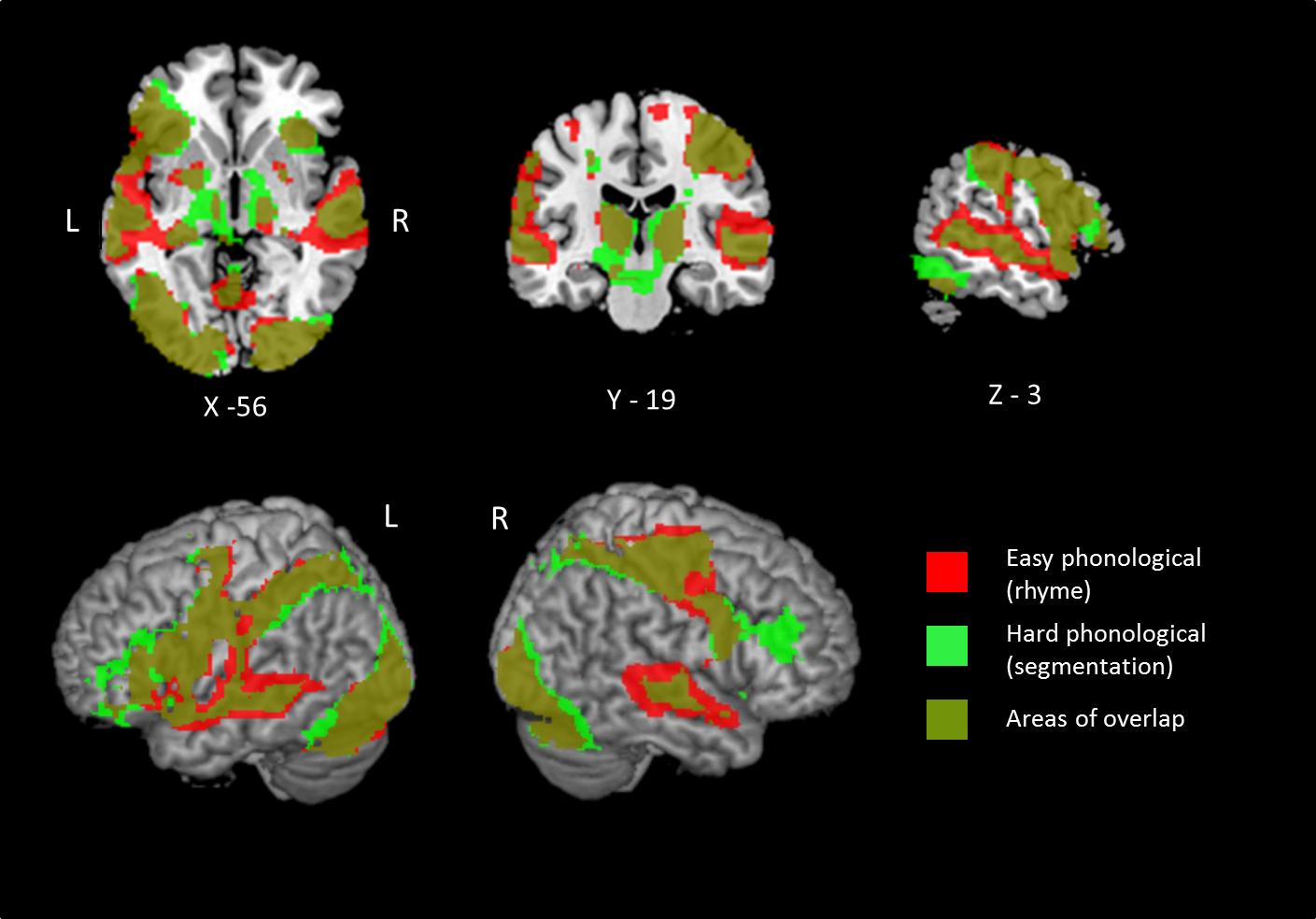


Figure S1: Whole brain analysis (cluster correction, Z > 2.3, p < .05), showing non-semantic tasks over rest. L = left, R = right hemisphere.


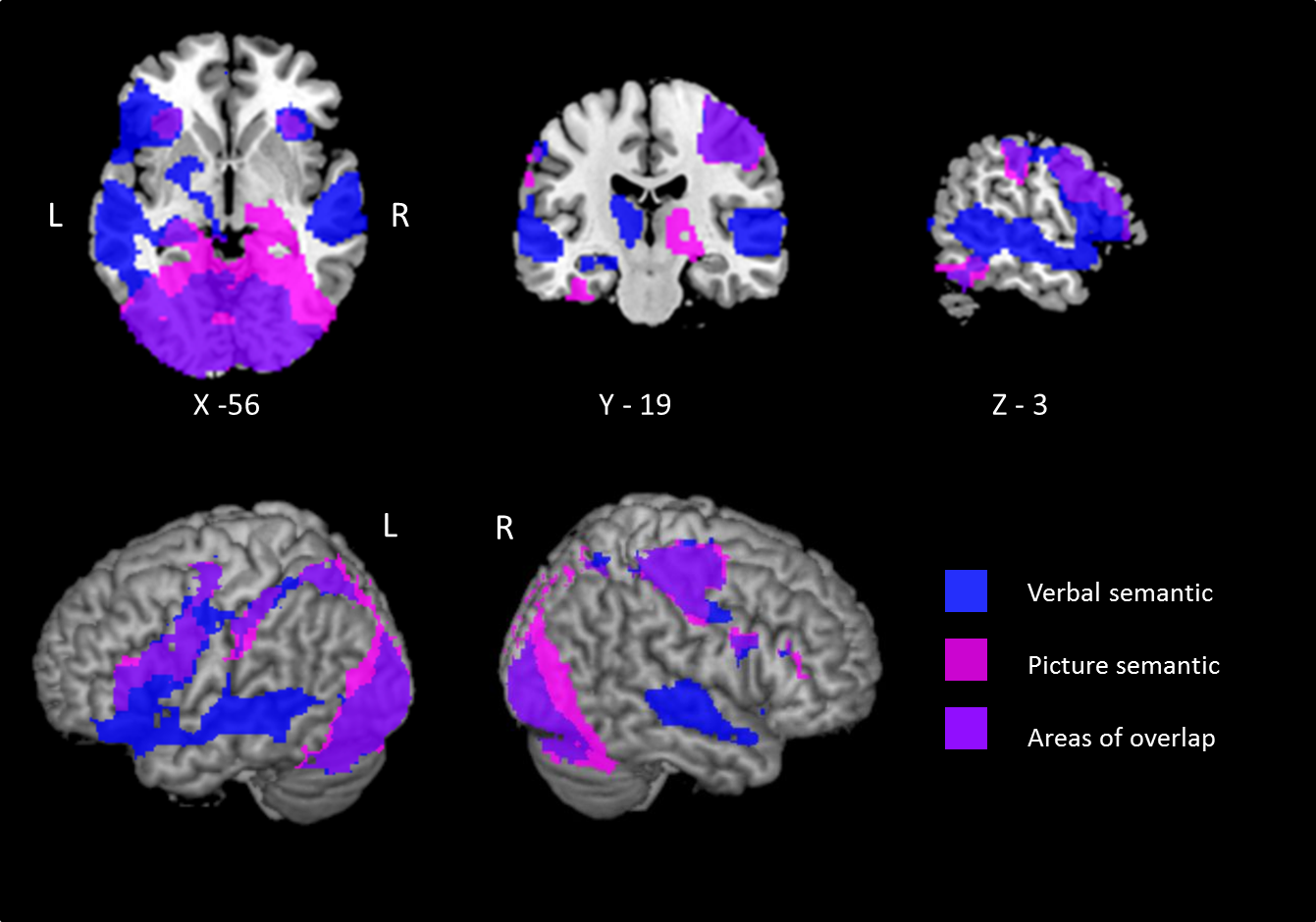


Figure S2: Whole brain analysis (cluster correction, Z > 2.3, p < .05), showing semantic tasks over rest. L = left, R = right hemisphere.


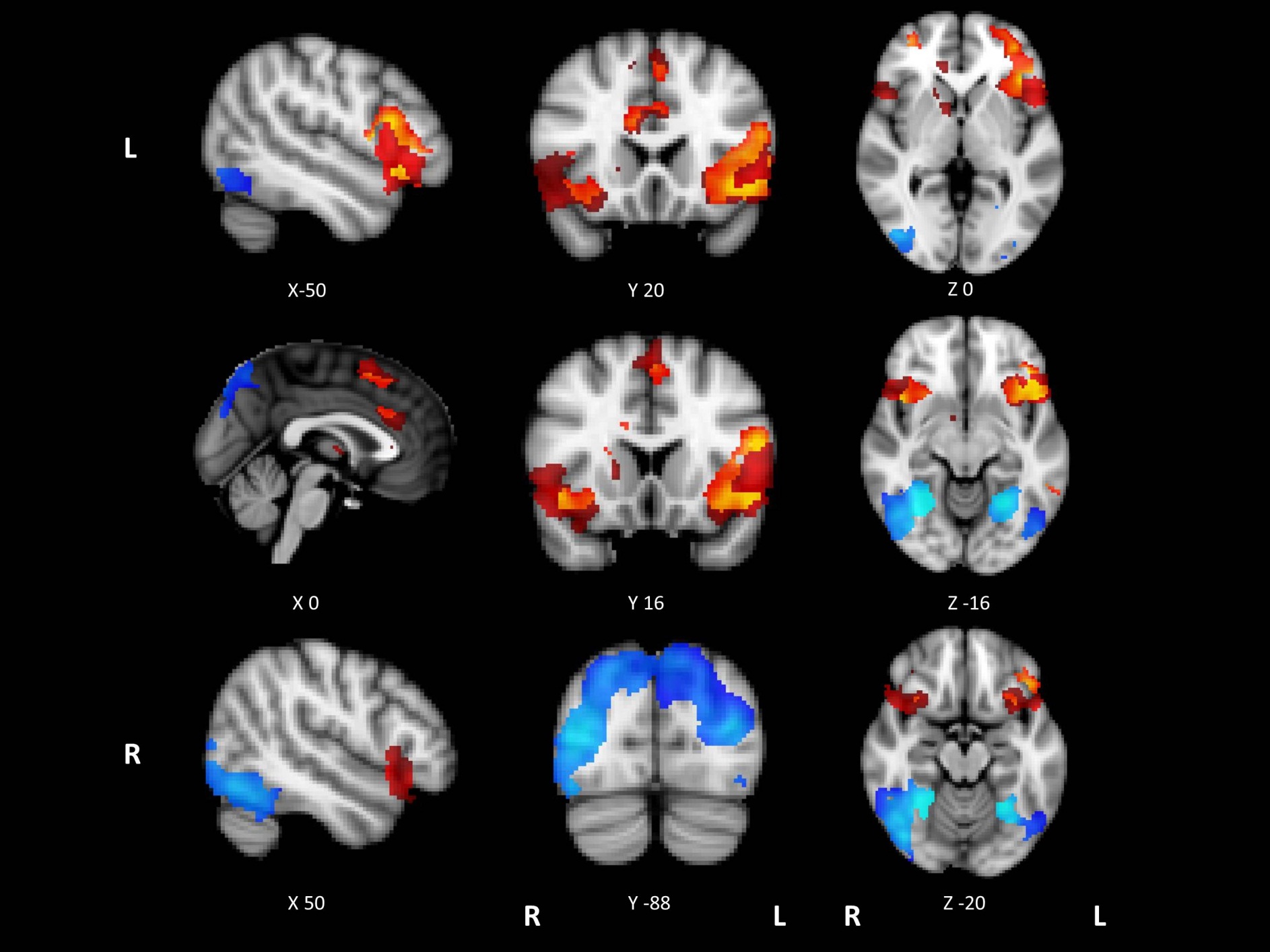


Figure S3: Effects of modality (verbal vs. picture semantic judgements) masked by voxels showing a significant response to difficulty.

Footnote to Figure S3: Effects of modality (i.e., voxels in which the response during a semantic task was significantly different for words vs. pictures), masked by two contrasts examining difficulty: (i) the contrast of semantic hard > easy and (ii) the contrast of non-semantic hard > easy. The areas in red/yellow correspond to voxels showing a greater response to verbal semantic judgements that were also sensitive to difficulty (orange/yellow = masked by semantic difficulty; red = masked by non-semantic difficulty). The areas in blue correspond to voxels showing a greater response to picture semantic judgements that were also sensitive to difficulty (masked by hard > easy for the non-semantic tasks). There were no clusters responding to pictures more than words that overlapped with difficulty within the semantic tasks. L = left, R = right hemisphere. All contrasts are cluster-corrected for whole-brain analysis (Z > 2.3, p < .05).

Figure S4: Interaction between difficulty (associative strength) and modality for semantic judgements.


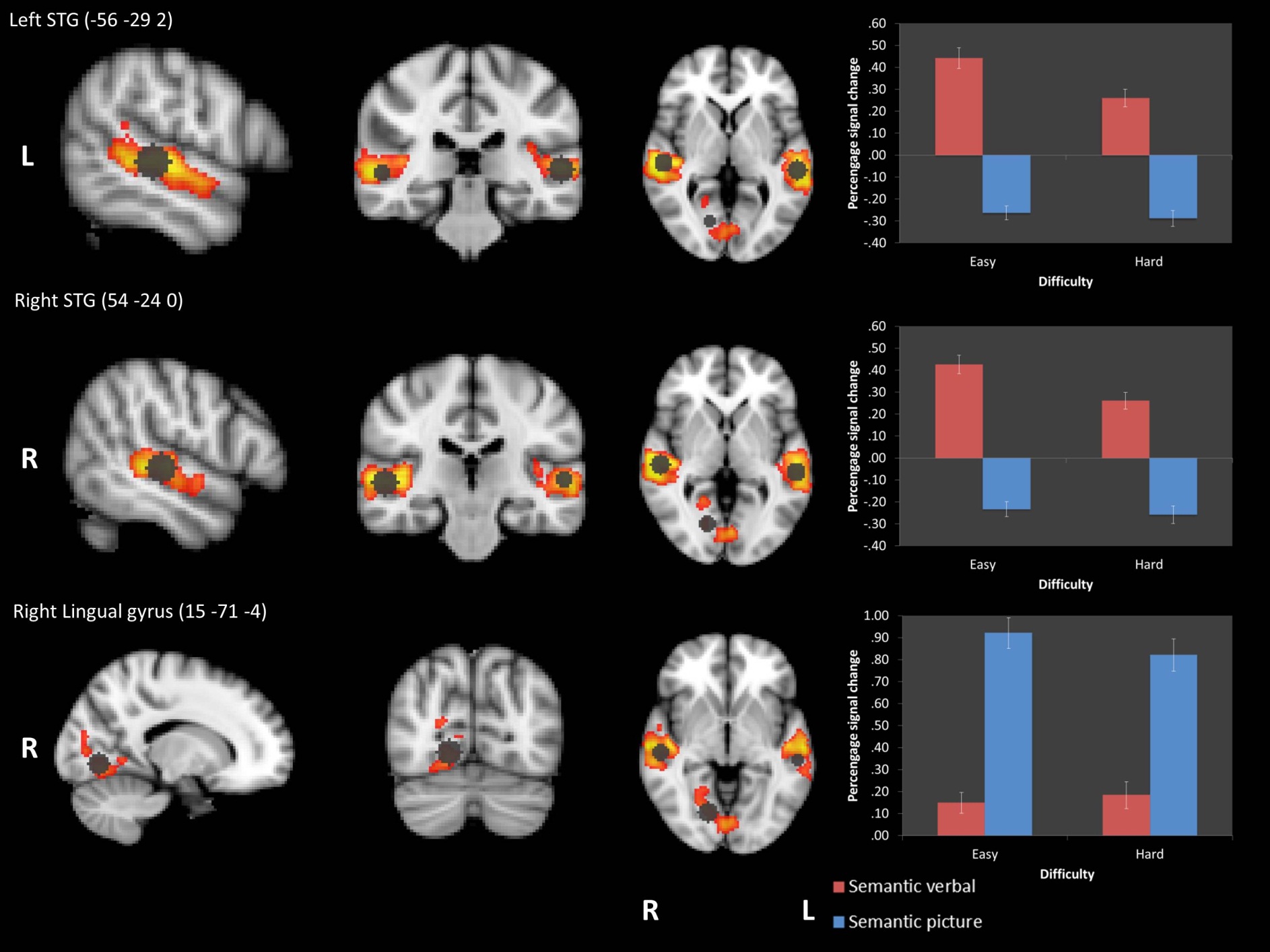


Footnote to Figure S4: Interaction between semantic task modality (words vs. pictures) and difficulty (easy: high associative strength vs. hard: low associative strength between probe and target). The interaction was computed at the higher level. Statistical maps are presented on the MNI-152 standard brain and are corrected for multiple comparisons (Z = 2.3, cluster correction applied, *p* < .05). Grey circles represent the locations of 8mm spheres placed around the centre of gravity for each cluster and used to investigate the nature of the interaction at each site. For the voxels within these spheres, FEATquery was used to extract percentage signal change for each of the conditions, represented in the graphs at the right of the figure. L = left, R = right hemisphere.
